# Assessing value of innovative molecular diagnostic tests in the concept of predictive, preventive, and personalized medicine

**DOI:** 10.1186/s13167-015-0041-3

**Published:** 2015-09-30

**Authors:** Ildar Akhmetov, Rostyslav V. Bubnov

**Affiliations:** Strategic Market Intelligence Dep., Unicorn, P.O.B. 91, Zhytomyr, 10020 Ukraine; Clinical Hospital “Pheophania” of State Affairs Department, Zabolotny Str., 21, Kyiv, 03680 Ukraine; Zabolotny Institute of Microbiology and Virology, National Academy of Sciences of Ukraine, Zabolotny Str., 154, Kyiv, 03680 Ukraine

**Keywords:** Predictive Preventive Personalized Medicine, Market access, Value, Strategy, Companion diagnostics, Cost-effectiveness, Reimbursement, Health technology assessment, Economic models

## Abstract

Molecular diagnostic tests drive the scientific and technological uplift in the field of predictive, preventive, and personalized medicine offering invaluable clinical and socioeconomic benefits to the key stakeholders. Although the results of diagnostic tests are immensely influential, molecular diagnostic tests (MDx) are still grudgingly reimbursed by payers and amount for less than 5 % of the overall healthcare costs. This paper aims at defining the value of molecular diagnostic test and outlining the most important components of “value” from miscellaneous assessment frameworks, which go beyond accuracy and feasibility and impact the clinical adoption, informing healthcare resource allocation decisions. The authors suggest that the industry should facilitate discussions with various stakeholders throughout the entire assessment process in order to arrive at a consensus about the depth of evidence required for positive marketing authorization or reimbursement decisions. In light of the evolving “value-based healthcare” delivery practices, it is also recommended to account for social and ethical parameters of value, since these are anticipated to become as critical for reimbursement decisions and test acceptance as economic and clinical criteria.

## Review

### The value-based approach is needed in economic models in predictive, preventive, and personalized medicine

Fundamental shifts in the healthcare paradigm, driven by the gradual transition to “patient-centered” value-based health service delivery, open new horizons to personalized medicine offering the potential to provide timely and cost-effective medical solutions to stratified patient subpopulations with predictable outcome margins. By using biomarkers as measurable indicators of predisposition to or severity of a disease state, personalized medicine helps in early detection, monitoring, assessment of risks associated with a disease, and guiding therapeutic decisions [1, 2].

Today, close to 50 % of the early-stage pipeline assets and 30 % of late-stage molecular entities of the pharmaceutical companies involve the use of specific biomarkers [3]. Moreover, molecular diagnostic tests (also molecular genetic testing or MDx), which drive the growth in personalized medicine by detecting and measuring proteins, nucleic acids, or metabolites variations, represent the fastest developing segment in the diagnostic (Dx) market enjoying healthy 10 % annual growth and promising to achieve USD 12.78 billion by 2018 [4].

With more than 26,000 diagnostic tests available for over 3600 genes in the USA alone, personalized medicine undoubtedly becomes one of the hottest areas in the global healthcare sector [5]. Yet, acceptance and adoption of personalized medicine by wider community within the global health system requires that all major stakeholders understand and are able to measure value offered by this practice, which is a herculean task considering the diversity of stakeholders’ needs, lack of a uniform assessment criteria, and often intangible multi-parameter nature of “value.”

Unlike therapeutics (Rx), which undergo three phases of clinical trials prior to marketing authorization and whose effect on patients can be quite straightforwardly demonstrated by patient-reported outcome measures, there is no clarity in the molecular diagnostics field on how much evidence is required to prove the value of a test. As a rule, most of diagnostic studies focus on accuracy and feasibility, which is a hardly enough prerequisite for better patient health or other downstream improvements [6, 7].

Having no direct evidence on patient outcomes, a diagnostic test is frequently looked upon as a non-value adding service, since it does not treat patients like a therapeutic but rather improves physicians’ decisions on a therapeutic intervention. Nevertheless, it is estimated that the results of diagnostic tests are immensely influential affecting around 60–70 % of all clinical decisions, although they still amount for only 4–5 % of healthcare costs [8, 9]. Such underestimation of diagnostic value leads to confined market access to safer and more effective therapies, poorer (often “non-value-based”) reimbursement, weaker intellectual property rights protection, underdeveloped incentivization systems for diagnostic innovations, as well as limited returns on investment vis-à-vis high R&D costs.

The purpose for this paper is to define the value of a molecular diagnostic test and to outline the most important components of value from miscellaneous assessment frameworks, which go beyond accuracy and feasibility, informing healthcare resource allocation decisions and facilitating the clinical adoption.

### Defining value of MDx

Molecular diagnostic testing has moved to the forefront of present-day clinical practice and is widely used in miscellaneous areas of healthcare including oncology, neuroscience, cardiology, infectious diseases, metabolic disorders, etc. The emergence of highly accurate and user-friendly diagnostic tools based on blood, cerebrospinal fluid, urine, and saliva, in addition to biopsied tissue, contributes to better acceptance and adoption of MDx in physician offices, clinics, and community health centers that recognize value in individualizing diagnosis and treatment [10, 11].

Fundamental shift in healthcare delivery is a harbinger of new evolving diagnostic testing needs, which force the already sophisticated and sensitive assays to develop into more portable, easy-to-use, cost-effective and less time-consuming platforms. Thus, the recently approved OraQuick ADVANCE™ test for a simple at-home use allows HIV patients to learn their status in just 20–40 min with 99.87 % specificity and 93.64 % sensitivity, clearly demonstrating the gradual transition from “laboratory-centered” to “user-centered” healthcare delivery [12]. The other study of myoglobin and troponin point-of-care testing showed 55–66 % decrease in time to obtain test results with 96.9 % sensitivity and 99.6 % NPV, when compared to central laboratory procedures, suggesting that the use of in vitro diagnostic kits in point-of-care testing in certain cases may be more time-saving and effective than conventional diagnostic approaches [13, 14].

It is also worth noting that rapid diagnostic testing is evidenced to reduce complications and avoid deaths among patients. For example, the data from three randomized controlled trials (RCT) carried out in Denmark demonstrated reduction in the relative risk of death from colorectal cancer (CRC) to less than 0.70 for subjects adhering to the screening [15]. Additionally, the use of AdvanDx’s PNA FISH™ testing demonstrated a 42 % reduction in mortality of patients with highly drug-resistant enterococcus faecium infections [16].

The studies of clinical and economic impact of MDx in the context of personalized medicine demonstrate univocal results that signify value of individualized approach to diagnosis and treatment. The report by AEI-Brooking Joint Center for Regulatory Studies projected that testing for variants in the CYP2C9 and VKORC1 genes guiding the initial dosing of warfarin could provide USD 1.1 billion in annual savings to the US healthcare system and prevent 17,000 strokes with 85,000 bleeding events [17]. Additionally, USD 604 million in annual savings would be realized if genetic tests limiting anti-epidermal growth factor receptor (EGFR) therapy to metastatic colorectal cancer patients with wild-type KRAS tumors are carried out [18].

Molecular diagnostic tests aid in guiding physicians’ decisions on treatment, which becomes even more topical in light of increasing heterogeneity of diseases, high drug attrition, and associated problems of over- and undertreatment, tightening up budgets of healthcare centers. For instance, genetic testing of breast cancer patients with Oncotype DX™ assay resulted in altered treatment of 44 % of individuals, demonstrating high accuracy and bringing high degree of certainty of the test value [19]. Along the same lines, there is evidence that diagnostic tests have contributed to 30–50 % reductions in direct hospital and outpatient charges by identifying key alterations in health status and facilitating modifications in therapeutic interventions to improve patient outcomes [8].

MDx also improve adherence, compliance, and willingness to undergo treatment or prevention by means of better prognosis of disease occurrence and prediction of the response to treatment.Thus, PreDx Diabetes Risk™ testing assesses the patient risk for getting type-2 diabetes over a 5-year period motivating patients to undertake preventive measures and to change their unhealthy lifestyles [20]. Similarly, the literature review of patient compliance based on genetic testing in breast and colorectal cancers, hamochromatosis, thrombophilia, smoking cessation, and obesity showed high increase in compliance rates associated with MDx [21].

High value of molecular diagnostic testing is observed in the pharmaceutical pipeline, as it facilitates discovery of biomarker-based therapies targeting disease causes instead of symptoms. It is evident that today, 50 % of all clinical trials conducted by pharmaceutical companies collect DNA from patients in order to facilitate biomarker development [22]. Moreover, is also known that biomarker-based diagnostics used in clinical trials can increase chances of regulatory approval [23, 24] and enhance prescription [25, 26].

In certain cases, MDx assist pharmaceutical companies to rescue licensing and reimbursement by providing more convincing arguments to regulatory authorities and insurers on clinical outcomes and cost-effectiveness through stratification of population. For example, Iressa® (gefitinib) withdrawn from the market after failing to prove survival benefit in phase 3 trials regained marketing authorization in Europe in combination with the EGFR mutation test [27]. Likewise, Herceptin® (trastuzumab) was not considered to be cost-effective by NICE and SMC initially in the large gastric cancer population; however, once the HER2 overexpression subgroup was defined, the decision has been changed to positive [28].

Finally, with advancements in bioinformatics and emergence of novel multi-omic databases, like OpenBEL, it is anticipated that systems diagnostics (SysDx), incorporating a wide series of biomarkers from different biological disciplines, can add substantial clinical and socioeconomic value by being easily scalable to address much larger groups of patients and by comprehensively breaking down a single complex disease into multiple targets with tailored treatment options.

To sum up, molecular diagnostics go far beyond trivial diagnosis, but instead are critical in identifying individual risk for developing a disease, prescribing safe and effective therapies, assessing response to a therapeutic intervention throughout the course of treatment, preparing viable disease management strategies, etc. Moreover, new generation MDx can add downstream value by virtue of their evolving characteristics such as greater accuracy, higher throughput, shorter testing time, simplicity, portability, cost-effectiveness, and so on.

### Assessing value of MDx

In view of the polysemic nature of “value” of MDx, there are numerous interpretations and ways to measure this concept depending on which model is used and from whose perspective the technology is being assessed. For instance, the “innovativeness” of MDx is likely to be of high value from a commercial standpoint but of lesser value to patients, physicians, or payers. Along the same lines, the benefit of an accurate diagnosis may be of high value to patients and doctors, although challenging to quantify and to validate for payers.

#### General value assessment frameworks

Porter’s Value-Based Healthcare (VBH) model places patient atop of the hierarchical pyramid of significance, arguing that the true value of any healthcare service, including diagnostic testing, can only be conceived through a prism of the total bundle of products and services delivered to an individual patient over a cycle of care by correlating the final patient outcomes with the associated costs [29]. In other words, the only way to adequately assess the real value of a diagnostic test is to consider it as an integral part of the combined “efforts” of all links in the value chain involved in the process of delivering value to a patient (e.g., healthcare providers, caregivers, manufacturers, pharmacists, and laboratories), assigning weights to each link based on its contribution to the overall patient outcome and measuring the total costs required to deliver this outcome:

$$ \mathrm{Value}=\frac{{\mathrm{Patient}\ \mathrm{outcomes}}^{*}}{\mathrm{Costs}\ \mathrm{of}\ \mathrm{delivering}\ \mathrm{the}\ \mathrm{outcomes}} $$

*The patient outcomes in this model include prevention of illness; early detection; right diagnosis; right treatment to the right patient; rapid cycle time of diagnosis and treatment; treatment earlier in the causal chain of disease; less invasive treatment methods; fewer complications; fewer mistakes and repeats in treatment; faster recovery; more complete recovery; greater functionality and less need for long-term care; fewer recurrences, relapses, flare ups, or acute episodes; reduced need for ER visits; slower disease progression; less care-induced illness; etc. [30].

Despite implementation difficulties, which are caused by the need in substantial structural changes in healthcare systems and stakeholders’ willingness to collaborate and to step out from the comfort zone, this model has already been accepted by a number of American health centers (e.g., Cleveland Clinic [31], Joslin Diabetes Center [30], and Catawba Valley Medical Center [32]) and won recognition of a large insurance association in the USA (e.g., the Blue Cross Blue Shield of Michigan’s Value-Based Contract model [33]). The emergence of novel VBH practices, such as accountable care organizations (ACOs) and patient-centered medical centers (PCMCs), may lead to new levels of data collection capturing value of diagnostic tests in conjunction with miscellaneous treatment options, admissions, surgeries, and other healthcare services [34].

Another approach to assess value of a diagnostic test was introduced by Fryback and Thornbury [35], who proposed a hierarchical six-level evidence framework of evaluating diagnostic technologies, later developed by Silverstein and Boland [36], Mackenzie and Dixon [37], Pearson et al. [38], and di Ruffano et al. [6], including the following:

- ▪ Technical characteristics (e.g., testing time, portability, ease to interpret results)
- ▪ Diagnostic accuracy (e.g., sensitivity, specificity, area under the receiver operating characteristic curve)
- ▪ Impact on diagnostic thinking (e.g., % of cases in which a physician is confident that the test changes the diagnosis)
- ▪ Impact on therapeutic actions (e.g., % of cases in which the choice of treatment is changed after information from the test is provided, shorter time to treatment, improved adherence/willingness to take Rx)
- ▪ Impact on patient outcomes (e.g., differences in mortality, morbidity, or quality of life between patients managed with the test and those managed without it, decreased re-hospitalizations)
- ▪ Impact on societal outcomes (e.g., cost-effectiveness of an improvement in patient outcomes, such as cost per life-year saved, calculated from a societal perspective) [6, 35-38]

Harris et al. [39] complemented the above-mentioned framework outlining the necessity in measuring adverse effects or changes in their incidence related to diagnostics and the associated therapeutics [39]. Additionally, the scholars argue that both *immediate patient outcomes* (e.g., blood pressure, glucose levels, or malignant neoplasm regression) and *overall health outcomes* (e.g., incidence of heart attacks, diabetes progression, or cancer survival) should be reflected in the assessment model because surrogate markers alone (e.g., reduction in protein levels) may not be enough criteria to justify reimbursement or regulatory approval.

Apart from the criteria specified by Fryback and Thornbury [35] and their successors, Ferrante di Ruffano et al. [6] also recommended using two additional parameters to assess value—*feasibility* (e.g., acceptability, clinical contradictions, and failure rates) and *test process* (e.g., procedural harms or benefits, placebo effect) [6].

According to Harris et al. [39], Trikalinos et al. [40], and Ferrante di Ruffano et al. [6], one of the most important ways to provide real evidence for value of an innovative diagnostic test is to either conduct RCTs or to construct decision analysis models that combine sufficient sources of evidence [6, 39, 40]. Considering the high cost of direct studies, the scholars advise to validate intermediate outcomes or surrogate markers as predictive of patient outcomes, which are sufficient in many cases for third-party payers to provide reimbursement.

The value assessment framework developed by the “Grading Quality of Evidence and Strength of Recommendations for Diagnostic Tests and Strategies” (GRADE) Working Group [41] also encourages the use of observational studies to compare alternative diagnostic strategies and assess direct patient-important outcomes in those cases, where RCTs are not feasible to implement [41]. The GRADE Working Group advices that the data set should provide enough evidence on decreasing false negatives or false positives, increasing true positives or true negatives, high accuracy of patient classification vis-à-vis alternate tests, and improved outcomes of both affected and healthy patients. Moreover, if there is no available effective treatment linked to the test, the accurate diagnostic assay may still be beneficial, once it reduces test-related side effects and anxiety by avoiding wrong diagnosis or may contribute to prevention and healthy lifestyle of patients by means of prognostic or predictive information.

Hayes et al. [42] and Simon et al. [43] provide clear guidelines on how to select efficient indirect “prospective–retrospective” designs based on archived specimens for establishing the medical utility of a prognostic or predictive biomarker as an alternative to costly RCTs. These scholars proposed five levels of evidence (LOE) to determine the clinical utility of a tumor marker and to report the results of marker research, depending on the study design (A—“prospective,” B—“prospective with archived specimen,” C—“prospective/observational” and D—“retrospective/observational”) and the variables involved (e.g., trial type, patient data, collection and storage of specimen, and statistical analysis and validation) [42-44].

**Fig. 1:**
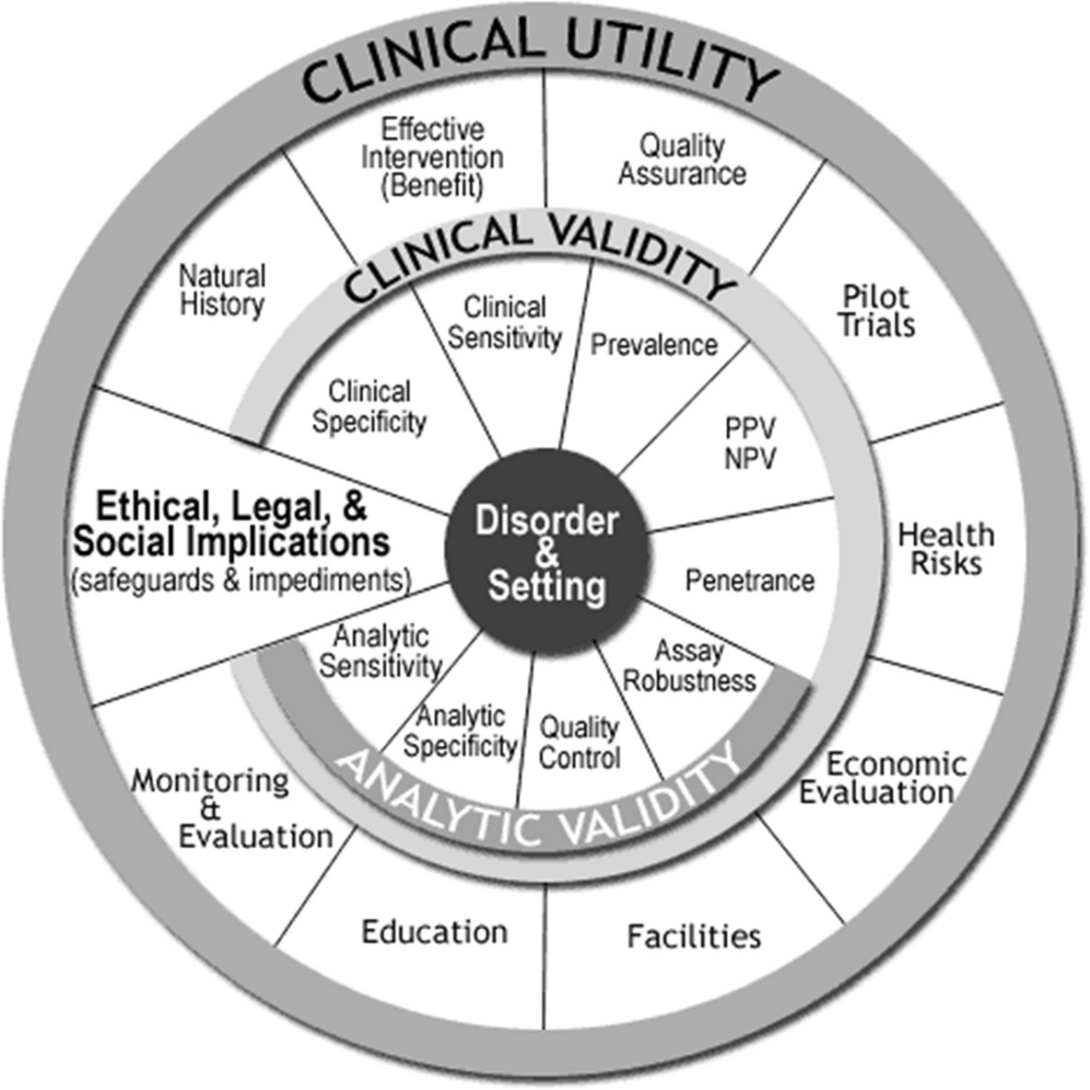
Graphic representation of the ACCE system for assessing genomic tests (adapted from Haddow and Palomaki [46])

Building up on Wald and Cuckle [45] framework and using terminology introduced by the Secretary’s Advisory Committee on Genetic Testing, Haddow and Palomaki [46] developed the ACCE model to evaluate genomic tests based on 44 targeted questions grouped into five categories: (1) clinical disorder and setting in which DNA testing is to be applied, (2) analytic validity, (3) clinical validity, (4) clinical utility, and (5) ethical, legal, and social implications (Fig. 1) [46]. This model differs from the aforementioned frameworks by addressing several important contextual issues impacting value of a MDx, like availability of facilities and personnel, robustness of education materials, methods for long-term monitoring, discrimination/ stigmatization, privacy, legal concerns, consent, ownership of data and/or samples, patents, licensing, proprietary testing, obligation to disclose, or reporting requirements, etc. [46].

Haddow and Palomaki [46] and National Academy of Sciences [44] further clarify that *clinical validity* is the association of a test result with an outcome (e.g., identifying disease and predicting adverse events) expressed by sensitivity, specificity, predictive values, odds and risk ratios, and logistic regression analyses [44]. *Analytic validity* deals with the technical performance of the test—its accuracy, repeatability, and reliability over time or under the influence of external substances. As to *clinical utility*, it describes how the test affects patient management and outcomes vis-à-vis the usual care and thus elucidates the significance of the test for individual patient decision-making. While clinical and analytic validity is most of the time required for marketing authorization, it is a clinical utility that plays a key role in the vast majority of reimbursement decisions.

Addressing the health outcomes issue, Bossuyt and McCaffery [47] have introduced a broader classification of patient outcomes that unlike Fryback’s and Thornbury’s [35] parameters incorporates six groups of testing “effects”—clinical management effect, direct health effect, emotional effect, social effect, cognitive effect, and behavioral response to testing [47]. Lee et al. [48] and Kopits et al. [49] supported such a broad classification citing examples from the Alzheimer’s studies, where patients expressed a willingness to know early in life about the chances of contracting the disease recognizing high *emotional and social value* in long-term planning (e.g., choices on lifestyle, work, retirement, financial plans, and reproduction) [48, 49].

According to Bossuyt and McCaffery [47], such multivariate taxonomy of patient outcomes broadens data collection, helps to better assess value, and facilitates improvements in overall delivery of quality healthcare. However, considering time and resource limitation, it can hardly happen that a diagnostic company incorporates all of these parameters into a value proof case. For that reason, a timely engagement with stakeholders throughout the entire assessment process is a prerequisite to better comprehension of stakeholders’ expectations and maximization of the evidence quality.

Finally, according to Task Force on Community Preventive Services, one of the most important considerations in assessing value of a diagnostic test is the perspective from which the analysis is carried out that predefines the composition of costs and health outcomes included in the analysis [50]. For example, a study conducted from a government perspective is likely to account for only those costs and benefits experienced by the government and disregard costs or benefits relevant to a health insurance purchaser, while value assessment from patient perspective is likely to incorporate much broader variety of costs (Table 1).

**Table 1:** Examples of costs included in a typical cost-effectiveness analysis based on the perspective of the analysis (adapted from Task Force on Community Preventive Services [50])

Cost	Perspective			
	Societal	Insurer/payer	Employer	Patient
Direct medical	Yes	Yes^a^	Yes^a^	Yes^b^
Direct non-medical (e.g., transportation, day care)	Yes	No	No	Yes
Indirect (e.g., time lost from work)	Yes^c^	No	Yes^c^	Yes^c^
Intangible (e.g., pain and suffering)	Yes^c^	No	No	Yes^c^

^a^Covered payments

^b^Out-of-pocket payments

^c^If not incorporated in the effect measure

The table suggests that while the societal and patient perspectives incorporate most of costs associated with a diagnostic test, payers’ and employers’ perspectives are much more selective. This proves the aforementioned statement that timely engagement with stakeholders may allow diagnostic companies to prepare more informed and custom-made evidence on financial impact of Dx testing with lesser time and money spent.

To sum up, generating direct and indirect evidence of causal relationships between a conducted test, improved health outcomes, and the associated costs is a convincing way to prove value of diagnostics. However, considering often nonlinear value chain of healthcare delivery, conducting such studies may be complicated, time-consuming, and costly. In view of the multi-parameter nature of value, it is recommended thereby to facilitate discussions with various stakeholders throughout the assessment process in order to arrive at a consensus about the depth of evidence required for positive regulatory or reimbursement decisions.

#### Private payer perspective

The growing influence of payers and the need of diagnostic producers to dispel payers’ “value-for-money” concern have endued the process of quantifying costs and outcomes in assessing value of molecular diagnostic tests with even greater importance. Currently, there is no uniform method to measure economic value among private payers or state programs neither in the developed nor in fast-growing economies, which leads to a continuous disconnect in valuation of MDx in the eyes of payers and diagnostic companies.

Of traditional methods to assess economic value of diagnostic tests comparing costs and outcomes, there are two most widely used approaches—*cost-effectiveness analysis* or CEA (expressed in delta costs compared with delta health outcomes) and *cost-benefit analysis* or CBA (usually expressed purely in monetary terms). Additionally, *cost-utility analysis* (CUA), which is a subtype of CEA, measures all costs in monetary units and benefits in terms of quality-adjusted life years (QALYs). While CEA and CUA provide information as to whether a health technology maximizes the health of a population in the resource-limited setting (widely used by payers), CBA is rather focusing on maximization of social welfare in light of societal budget constraints [51]. Yet, the accuracy of either economic assessment method depends very much on what data is available, what assumptions researchers make, how cost/outcome parameters are selected and validated, etc.

In the recent survey among small and medium businesses dealing with health technology assessment (HTA), the interviewed firms accepted that the main impediments in the reimbursement process for their products were poor understanding of specific payer requirements, insufficient scientific advices from the HTA bodies, lack of methodological agility and unnecessary bureaucracy resulting in belated identification of genetic variants, inadequate description of clinical trial designs, incomplete representation of patient experiences, etc. [52]. Additionally, the review of scientific articles containing evidence to help guide decision-making about insurance coverage for Alzheimer’s disease diagnostic tests, conducted by the Institute for Clinical and Economic Review (ICER) and Policy Development Group (PDG), showed that all studies without exclusion failed to provide convincing evidence that payers could use to showcase improved outcomes [53]. None of these studies established analytic validity by capturing action based upon diagnosis, health outcomes (e.g., cognitive/function decline), societal outcomes (e.g., cost-effectiveness), or technical efficacy [53].

As a result, a great number of companion MDx developed separately from therapeutics did not receive wide payer acceptance due to poorly established links between testing, therapeutic interventions, and health outcomes (e.g., tests to estimate warfarin dosage or CYP2C19 assays to stratify clopidogrel-eligible subpopulation) [54]. Additionally, according to the literature review by Paci and Ibaretta [55], 27 % of the assessed Dx tests failed to demonstrate favorable and univocal cost-effectiveness evidence compared to the standard of care [55].

Of the evaluation frameworks commonly cited by the third-party payers, each has a different approach to technology assessment, therefore creating inconsistencies in reimbursement decisions (Table 2).

**Table 2:** Coverage inconsistencies in molecular diagnostics (adapted from Gustavsen et al. [56]

Innovative test example	Positive coverage policies			
	Aetna	Regional CMS	Cigna	Regional BCBS
AlloMap™		X		
OncoType Dx™ (breast cancer)	X	X	X	X
MammaPrint™		X		
BRACAnalysis™	X	X	X	X
OVA1™		X		X
KRAS (ovarian cancer)	X	X	X	X

The table illustrates how conflicting the coverage decisions of leading US insurers may be with respect to the same molecular diagnostics. While some molecular assays have been included in the coverage list by all major payers as of 2010 (e.g., Oncotype Dx™, BRACAnalysis™, and the cobas® KRAS mutation test), other tests have only got positive coverage decisions by one or two payers (e.g., like AlloMap™, MammaPrint™, and OVA1™). This could be partially explained by the fact that in the USA, some payers use up to seven assessment frameworks to reason their reimbursement decisions (e.g., BCBS TEC, Hayes, ECRI, EGAPP, ICER, USPSTF, and Up-To-Date), while others give preferences to only one or two. Such discrepancies in assessment approaches and coverage policies create unnecessary confusion for manufacturers misguiding physicians and patients on the clinical value of molecular diagnostic tests.

The lack of consistency in value demarcation has pushed the ICER to release a “value framework” draft, which is set to streamline the assessment processes from payer perspective and to facilitate more structured discussions between various stakeholders around value of new health technologies.

The framework suggests that apart from comparative clinical effectiveness measuring the degree of the comparative net health benefit versus costs mentioned above, the assessment process must also incorporate contextual considerations enhancing the value of diagnostics and favoring more informed reimbursement decisions. For example, it makes a huge difference for public and private insurers, whether they are dealing with an *innovative technology* or there is already an alternative existing on the market (e.g., new biomarkers and different mechanisms of action) [57]. Amidst other contextual considerations, which increase the value of a MDx in the eyes of payers, are the following:

- ▪ High severity of the condition (e.g., neurodegeneration and oncology);
- ▪ High vulnerability of the population (e.g., pregnant women and children);
- ▪ Ability to expand the eligible population for treatment (e.g., higher sensitivity/specificity);
- ▪ Ability to improve the delivery system (e.g., preparation, storage, or delivery of the therapy);
- ▪ Ability to decrease the misuse or overuse of therapies (e.g., precise drug dosage, over/under diagnosis, over-/undertreatment);
- ▪ Low risk of pushback from precedents (e.g., other payer coverage decisions);
- ▪ Low risk of pushback from clinicians and provider groups if not preferred (e.g., high complexity of tests), etc. [57, 58]

The ICER framework is one of the first attempts to convince payers to integrate cost-effectiveness thinking into the way that they actually conduct their assessments in a more coherent way, and to encourage development of new policy tools to attain high health system value in conjunction with high clinical care value [58]. However, without clear guidelines on quantification of value of diagnostic tests, it may hardly occur that reimbursement authorities arrive at a consistency in the coverage decisions on their own.

#### State perspective

In light of the nonhomogeneous global economic and political environment, significant differences in HTA and reimbursement are observed at a state level, predefining the diversity of reimbursement and budgetary allocation practices. Some countries, like the UK, rely on cost-utility analysis in their reimbursement decisions by evaluating the cost of interventions versus the obtained health benefit (e.g., QALY). Other countries, like France and Germany, consider clinical added value with subsequent “value for money” pricing debates.

High variability of approaches to assess cost-effectiveness of molecular diagnostics can be exemplified by EGFR testing before Iressa® (gefitinib) trial. While the MDx producer’s submission estimated cost-effectiveness of the test at GBP 23,615 per QALY, the assessment conducted by NICE demonstrated GBP 35,700 per QALY and the study by SMC measured cost-effectiveness as GBP 154,022 per QALY [54]. This signifies the need for better modeling tools and coherent methodological frameworks to assess economic outcomes of MDx strategies.

The diversity in HTA systems is further complicated by centralized versus decentralized reimbursement decision-making. In most of countries, coverage for prescription medicines is done at the national level, while diagnostic manufacturers have to deal with local payers in order to get reimbursement for their clinical diagnostic (CDx) tests. For example, despite of the fact that trastuzumab is reimbursed in most of European member states, its HER2/neu companion diagnostic test is publicly funded in the UK, Germany, and Italy, while in Spain, it is covered by a therapeutic partner [59].

Countries employ miscellaneous analytical frameworks to guide their evaluations assessing multiple criteria like clinical effectiveness, safety, costs of a technology versus its benefits, etc. (Table 3).

**Table 3:** Criteria for health technology assessment in Europe (adapted from Sorenson et al. [60])

Criteria	AT	BE	CH	DE	FI	FR	NL	NO	SE	UK
Clinical benefit	X	X	X	X	X	X	X	X	X	X
Patient benefit	X	X	X	X	X	X	X	X	X	X
Cost-effectiveness	X	X			X		X	X	X	X
Budget impact		X			X	X	X	X		X
Innovative characteristics	X	X				X	X			X
Availability of alternatives	X						X		X	X
Equity considerations								X	X	X
Public health impact						X				
R&D					X					

Reproduced with permission from the European Observatory on Health Systems and Policies

*AT* Austria, *BE* Belgium, *CH* Switzerland, *DE* Denmark, *FI* Finland, *FR* France, *NL* Netherlands, *NO* Norway, *SE* Sweden, *UK* United Kingdom

While it is a policy in question that predetermines the use of a particular analytical framework, the vast majority of public assessment models account for clinical and patient benefit in the first place. Moreover, the evolving role of societal perspective in the evaluation process is shaping the assessment mechanisms resulting in the fact that today, some countries start considering costs and benefits outside the health sector as additional criteria for assessing value. For example, the Working Group 4 report introduced by Busse et al. [61] supported the idea that assessment should go beyond safety, efficacy/effectiveness, and economic aspects accounting for other meaningful outcomes, like the following:

- Psychological/social/ethical outcomes (e.g., compliance, acceptance, satisfaction, demand, preferences, and information/patient advice requirements)
- Organizational/professional outcomes (e.g., utilization of service, change in the treatment location, change in length of hospital stay, change in required personnel, material inputs, and training requirements) [61]

To sum up, the heterogeneity of approaches to assess value of an innovative health technology, including MDx, create significant market access hurdles for innovative Dx tests. Therefore, joined efforts among various stakeholders are required to shape the way assessment is carried out in different healthcare systems. This could be achieved by means of a common language and terminology, uniform HTA assessment frameworks, more transparent and comprehensive reporting of the findings, etc.

### Case studies

In the history of personalized medicine, there have been numerous examples of molecular tests receiving positive and negative reimbursement decisions by private and public payers in the USA and Europe. Below are several case studies from miscellaneous therapeutic areas which briefly describe coverage decisions for the selected molecular diagnostic tests.

#### Oncology—breast cancer

Dako’s immunohistochemistry assay HercepTest™ is one of the brightest examples of the successful application of molecular diagnostics in oncology by targeting HER2/neu overexpression in metastatic breast cancer. The test enabled stratification of breast cancer patients into eligible and non-eligible for Roche/Genentech’s monoclonal antibody therapeutic Herceptin® (trastuzumab) based on the prognostic biomarker HER2/neu presented in 20–30 % of breast cancer patients [62, 63]. The reimbursement decisions for the HER2/neu companion diagnostic were largely based on the results of RCTs, cost-effectiveness, and the ability to link the test to QALYs gained from treating the stratified subpopulation receiving trastuzumab.

While Herceptin® is commonly reimbursed in most of the EU member states, no single test has been accepted globally as the gold standard for measuring HER2/neu overexpression since 1998. Thus, in the UK, Germany, and Italy, HER2 test is covered by the state, while in Spain, it is an Rx partner who pays for testing. In France, HER2 test was authorized in 2000 but received positive reimbursement decision only in 2007 [64].

#### Oncology—colorectal cancer

Approximately 30–50 % of colorectal tumors are associated with an abnormal KRAS gene, signifying that nearly half of patients with colorectal cancer (CRC) might respond to anti-epidermal growth factor receptor (EGFR) treatment, and the other half might not [65]. Two monoclonal antibodies, Erbitux® (cetuximab) and Vectibix® (panitumumab), have demonstrated favorable survival impact in population with KRAS wild-type CRC [66, 67]. Hence, KRAS mutation testing is currently used in clinical practice to assist in identifying eligible patients for antibody treatment.

The positive reimbursement decisions of the majority of American and European payers were primarily based on the strong linkage between the clinical utility of the test and patient outcomes derived from the post factum analysis of cetuximab and panitumumab clinical trial data [68-70], cost-effectiveness [71], and inclusion of the test in clinical practice guidelines [72]. This is one of the first examples of diagnostic companies receiving wide payer acceptance based solely on retrospective archived samples data without conducting heavy and costly RCTs.

Other examples of successful reimbursement of molecular diagnostics in oncology include Oncotype DX®, MammaPrint®, and OncoVue® (breast cancer); EGFR mutation assays (non-small cell lung cancer); BCR-ABL testing (chronic myelogenous leukemia); and others.

#### HIV

The HLA-B*5701 molecule is associated with hypersensitivity to the antiretroviral drug abacavir; hence, a positive test allows physicians to use the drug in a much larger population due to the assay’s ability to identify patients that are at risk of developing severe adverse events [73].

The reimbursement decisions for the HLA-B*5701 testing were primarily based on data from RCTs and retrospective studies, cost-effectiveness, and tests’ ability to identify broader population of patients with high degree of sensitivity and specificity. The cost of the test was justified by clear clinical utility (prospective and retrospective studies showing improved health outcomes [74]), cost benefits (including management of the adverse event [73]), and cost-effectiveness (e.g., incremental cost-effectiveness ratio per hypersensitivity reaction avoided [75]). Additionally, inclusion of abacavir into the treatment guidelines along with the companion diagnostic has reinforced reimbursement of HLA-B*5701 test by private and public payers [76].

#### Hepatitis C

Viral load monitoring (VLM) is a quantitative PCR or bDNA test used by physicians to confirm the presence of HCV and to measure the patient’s viral load with hepatitis C. Despite the fact that VLM does not predict the development of cirrhosis or liver failure, like the aforementioned HIV testing tools, it shows the likelihood of patient’s response to treatment.

The ability to predict therapeutic outcomes, avoiding costs associated with treatment of non-respondents and minimizing adverse effects associated with the intervention, laid the basis for the test to be include in the reimbursement lists by payers in the USA and Europe [77].

#### Cardiovascular

CardioDX’ Corus® CAD is a multi-analyte gene expression assay introduced as a less invasive way to identify obstructive coronary artery disease (CAD) and assist primary care physicians and cardiologists to understand whether the symptoms of cardiovascular disease in non-diabetic patients are caused by CAD.

Seeking for wide payer acceptance, CardioDX has invested heavily into the three costly clinical utility studies (PREDICT, COMPASS [78], and IMPACT-PCP [79]) and received Medicare reimbursement via the Palmetto MolDx program based on reduced costs by avoiding coronary angiography and other invasive assays [80]. However, of the 10 largest private payers in the USA, Aetna Health was the only organization that included the test into its coverage list, while the rest of payers rejected Corus® CAD because it had a poorly established link between clinical decision-making and patient outcomes [81].

This case study signifies that massive prospective studies, which lay the ground for clinical utility, are important, but not the only critical point in a reimbursement decision; therefore, companies have to carefully account for other payer requirements with regard to economic, clinical, and social value of molecular diagnostics, in order to get reimbursement and wider test acceptance.

## Conclusions

Innovative molecular diagnostics are becoming an essential part of disease management and therapy, helping physicians to stratify patient cohorts, choose more appropriate drug regimen, avoid adverse events, facilitate therapeutic monitoring, and define the predisposition to a disease. Notwithstanding their importance, MDx are still frequently perceived as “additional costs” and get much lower reimbursement rates and profit margins than therapeutics. However, in the nearest term, the new generation molecular assays are expected to alter this status quo, either by becoming “companion diagnostics” or by demonstrating substantial value, like cost reduction through targeted treatment, reduction in the number of side effects, etc.

Due to the polysemic nature of “value,” there are many ways to assess MDx depending on which framework is used and from whose perspective the technology is being evaluated. Thereby, it is recommended for industry to facilitate discussions with various stakeholders throughout the assessment process in order to arrive at a consensus about the depth of evidence required for positive regulatory/reimbursement decisions and clinical adoption, gazing beyond accuracy and feasibility data. It is also recommended to develop clear guidelines on quantification of value of diagnostic tests to avoid inconsistency in the coverage decisions by public and private payers.

The further lines of our research will address the following topics: (1) the impact of social and ethical parameters of value on reimbursement and test acceptance in light of the evolving “value-based healthcare” delivery practices; (2) the evolving innovative trial designs driven by smaller MDx-based studies with stratified patient cohorts and archived specimen, contributing to level I evidence and replacing massive RCTs; and (3) investigation into the heterogeneity of the state-level HTA frameworks.

## Abbreviations

CDx: clinical diagnostic
Dx: diagnostic
MDx: molecular diagnostic tests
